# Effect of Mulberry Leaf and Its Active Component, 1-Deoxynojirimycin, on Palmitic Acid-Induced Lipid Accumulation in HepG2 Cells

**DOI:** 10.3390/biomedicines13122930

**Published:** 2025-11-28

**Authors:** Dahae Lee, Jiyeon Kim, Min Ji Han, Seon Hwa Kim, Tae Hoon Kim, Dae-Woon Eom, Inhyeok Song, Daesik Jeong, Noriko Yamabe, Ki Hyun Kim

**Affiliations:** 1 School of Pharmacy, Sungkyunkwan University, Suwon 16419, Republic of Korea; pjsldh@gachon.ac.kr (D.L.);; 2 Vixxol Corporation, Gunpo 15807, Republic of Korea; minjhan@vixxol.com (M.J.H.); seonhkim@vixxol.com (S.H.K.); andykim@vixxol.com (T.H.K.); 3 Department of Pathology, University of Ulsan College of Medicine, Gangneung Asan Hospital, Gangneung 210-711, Republic of Korea; edwjyh@hanmail.net; 4 Bio DX Group, InSiliCox, Seoul 03016, Republic of Korea; inhyeok@5works.co.kr (I.S.); jungsoft97@smu.ac.kr (D.J.); 5 Faculty of SW Convergence, Sangmyung University, Seoul 03016, Republic of Korea

**Keywords:** palmitic acid, 1-deoxynojirimycin, L-leucine, FAS, PPAR-γ, HepG2

## Abstract

**Objectives:** Aqueous mulberry leaf extract (MLE) contains 1-deoxynojirimycin (DNJ) and L-leucine (LL). This study investigated the effects of MLE, DNJ, and LL on lipid accumulation caused by palmitic acid (PA) in human hepatoma HepG2 cells, pro-inflammatory cytokine levels, and regulation of lipogenesis. **Methods:** PA was applied to HepG2 cells to generate a fatty liver in vitro model. Then, the cells were treated with MLE, DNJ, or LL for 24 h. Western blot analysis was performed to determine the protein expression levels of peroxisome proliferator-activated receptor gamma (PPAR-γ) and fatty acid synthase (FAS) in HepG2 cells. **Results:** Staining with Oil Red O (ORO) indicated that MLE, DNJ, and LL significantly decreased excessive lipid accumulation in HepG2 cells. Cytokine ELISA assay indicated that MLE, DNJ, and LL significantly decreased excessive pro-inflammatory cytokine levels in HepG2 cells. In addition, MLE, DNJ, and LL decreased the protein expression levels of PPAR-γ and FAS, suggesting a potential suppression of lipogenesis. **Conclusions:** Our results suggest that MLE, DNJ, and LL reduce lipid accumulation, pro-inflammatory cytokine levels, and the protein expressions of FAS and PPAR-γ in PA-induced fatty liver cells.

## 1. Introduction

Non-alcoholic fatty liver disease (NAFLD) is caused by aberrant lipid accumulation in hepatocytes, which is brought on by inflammation and lipid accumulation in the liver. In NAFLD, lipid accumulation (hepatic steatosis) in the liver is induced by increased lipid synthesis and decreased glycogen synthesis. Inflammation results from this, and non-alcoholic steatohepatitis (NASH) develops if it continues [1]. Hepatic lipid production depends on transcription factors that control hepatic lipogenesis, such as peroxisome proliferator-activated receptor gamma (PPAR-γ), which initiates fatty acid chain synthesis, which triggers excessive triglyceride synthesis and lipid accumulation in the liver [2,3]. Also, PPAR-γ regulates inflammatory cytokine genes monocyte chemoattractant protein (MCP)-1 and interleukin (IL)-6 related to immune inflammatory response in the progression of NAFLD [4]. Saturated fatty acid promotes the IL-6 and MCP-1 and IL-6 activities, which exacerbate hepatic lipid synthesis and inflammation in NAFLD [5].

A commercially available water-based extract of the leaves of *Morus alba* Linn (*M. alba*) contains 1-deoxynojirimycin (DNJ) and L-leucine (LL). Recent studies have shown that water-based extract of mulberry leaves, as well as its active components, 1-deoxynojirimycin (DNJ) and L-leucine (LL), can improve NASH in STAM mice [6,7,8] and mitigate hyperlipidemia in high-fat diet-induced steatohepatitis in rats [9,10]. Another study identified that mulberry leaf extracts reduce plasma lipid profiles in males between 20–64 years of age, with a good safety profile for short-term use [11]. In this study, a palmitic acid (PA)-treated HepG2 model was chosen for anti-lipogenic/inflammatory assay. PA is a saturated fatty acid with a straight chain.

In this regard, evidence supports the notion that saturated fatty acids predispose the liver to excessive lipid accumulation and metabolic dysfunction [12]. We hypothesized that mulberry leaf extract (MLE), a commercially available water-based extract of mulberry leaves, reduces lipid accumulation and pro-inflammatory cytokine levels. Therefore, we evaluated the effects of MLE and its active components, DNJ and LL, in PA-treated HepG2 cells.

## 2. Materials and Methods

### 2.1. Chemicals and Reagents

The aqueous mulberry leaf extract (MLE) used in this study was manufactured by Phynova Group Ltd. (Long Hanborough, UK) and distributed by Vixxol (Gunpo, Republic of Korea). The extract is standardized to contain 5% (±10%; 4.5–5.5%) 1-deoxynojirimycin (DNJ) [13,14]. Batch-to-batch consistency is maintained through a quality control process that begins with raw mulberry leaf selection to ensure a minimum DNJ content. Production typically yields batches with >5% DNJ, and final standardization is achieved through batch blending and dilution with excipients. Each batch is subjected to rigorous quality control, including quantitative DNJ analysis using HPLC–ELSD and qualitative fingerprinting using HPTLC. LC/MS chromatographic analysis further confirmed the presence of DNJ and identified L-leucine (LL) in the extract (Figure S2), although LL is not included as part of the standardized content. DNJ and LL, used as standards in this study, were purchased from Sigma-Aldrich (St. Louis, MO, USA).

### 2.2. Cell Culture

The human hepatocellular carcinoma cell line HepG2 was obtained from the American Type Culture Collection (ATCC, Manassas, VA, USA). Cells were cultured in Dulbecco’s Modified Eagle’s Medium (DMEM; Cellgro, Manassas, VA, USA) supplemented with 10% fetal bovine serum (FBS; Gibco, Grand Island, NY, USA) and 1% penicillin–streptomycin (Invitrogen, Grand Island, NY, USA). Cultures were maintained at 37 °C in a humidified incubator containing 5% CO_2_ and 95% air. The medium was replaced every 2–3 days, and cells were subcultured at approximately 80–90% confluence using 0.25% trypsin–EDTA solution (Gibco). HepG2 cells at passages 20–25 were used for all experiments [15].

### 2.3. Palmitic Acid-Induced Lipid Accumulation in HepG2 Cells

HepG2 cells were seeded into 96-well plates at a density of 1 × 10^4^ cells/well and allowed to adhere overnight at 37 °C in a humidified atmosphere containing 5% CO_2_. The cells were then pretreated with or without DNJ, LL, rosiglitazone, or MLE for 2 h, followed by exposure to 0.5 mM PA for 24 h to induce lipid accumulation. After treatment, the cells were washed twice with phosphate-buffered saline (PBS) and fixed with 10% (*v*/*v*) formalin for 30 min at room temperature. Following fixation, the cells were rinsed with 60% isopropanol and stained with freshly prepared Oil Red O (ORO) working solution (prepared by diluting the stock solution with distilled water at a ratio of 3:2) for 15 min. Excess dye was removed by washing with distilled water. The stained lipid droplets were visualized under an optical microscope, and intracellular lipid accumulation was quantified by eluting the dye with 100% isopropanol and measuring the absorbance at 500 nm using a microplate reader. The procedure was performed according to the method described in [16], with minor modifications.

### 2.4. Measurement of Pro-Inflammatory Cytokine Levels

The concentrations of interleukin-6 (IL-6) and monocyte chemoattractant protein-1 (MCP-1) in the cell culture supernatant were determined using commercial ELISA kits (R&D Systems, Minneapolis, MN, USA), following the manufacturer’s protocols and the method described in reference [17]. Briefly, after the indicated treatments, the culture medium was collected and centrifuged at 1000× *g* for 10 min at 4 °C to remove cell debris. The clarified supernatants were then transferred to 96-well ELISA plates pre-coated with specific capture antibodies for IL-6 or MCP-1. Standards and samples were added in duplicate, and the plates were incubated according to the kit instructions. After washing to remove unbound materials, biotin-conjugated detection antibodies were added, followed by incubation with streptavidin–horseradish peroxidase (HRP). The colorimetric reaction was developed by adding the tetramethylbenzidine (TMB) substrate, and the reaction was stopped with 2 N sulfuric acid. Absorbance was measured at 450 nm using a microplate reader. The concentrations of IL-6 and MCP-1 were calculated from standard curves generated using known concentrations of each cytokine.

### 2.5. Western Blotting Analysis

The target protein expression levels are determined using the method described in reference [18,19]. Protein expression was examined by Western blotting analysis. The HepG2 cells were seeded into a 6-well plate at a density of 8 × 10^5^ cells/plate with or without MLE, DNJ, or LL and incubated for 2 h, followed by incubation with PA (0.5 mM) for 24 h. Cells were then collected in a harvest medium (Dulbecco’s modified Eagle’s medium) and washed with phosphate-buffered saline. Cells were lysed using radioimmunoprecipitation assay buffer (pH 7.4; Cell Signaling Technology, Danvers, MA, USA). Protein concentrations were measured with the bicinchoninic acid (BCA) assay kit (Thermo Fisher Scientific, Inc., Waltham, MA, USA). The 20 μg of protein per lane was separated via 10% sodium dodecyl sulfate-polyacrylamide gel electrophoresis and transferred onto polyvinylidene difluoride membranes. Precision Plus Protein Unstained Protein Standards (Bio-Rad Laboratories, Inc., Hercules, CA, USA, #161-0363; range: 10–250 kDa) were used as molecular weight markers. Membranes were incubated with primary antibodies (1:1000 dilution; Cell Signaling Technology) against the FAS (#4233S), PPAR-γ (#2435S), glyceraldehyde 3-phosphate dehydrogenase (#2118S) overnight at 4 °C, followed by incubation with secondary antibodies (1:2000 dilution; Cell Signaling Technology). The membranes were treated with enhanced chemiluminescence plus Western blotting detection reagent (GE Healthcare, Little Chalfont, UK) and visualized using a chemiluminescence system (FUSION Solo; PEQLAB Biotechnologie GmbH, Erlangen, Germany). Protein band intensities for FAS and PPARγ were normalized to GAPDH and expressed relative to the untreated control.

### 2.6. Statistical Analysis

All analyses were conducted using SPSS Statistics ver. 19.0 (SPSS Inc., Chicago, IL, USA). Data are presented as mean ± SEM for clarity. Nonparametric comparisons were performed using the Kruskal–Wallis test due to the small sample size and non-normal distribution of the data. A *p*-value < 0.05 was considered statistically significant.

## 3. Results

### 3.1. Inhibitory Effects of MLE, DNJ, and LL on Lipid Accumulation in HepG2 Cells

**Figure 1:**
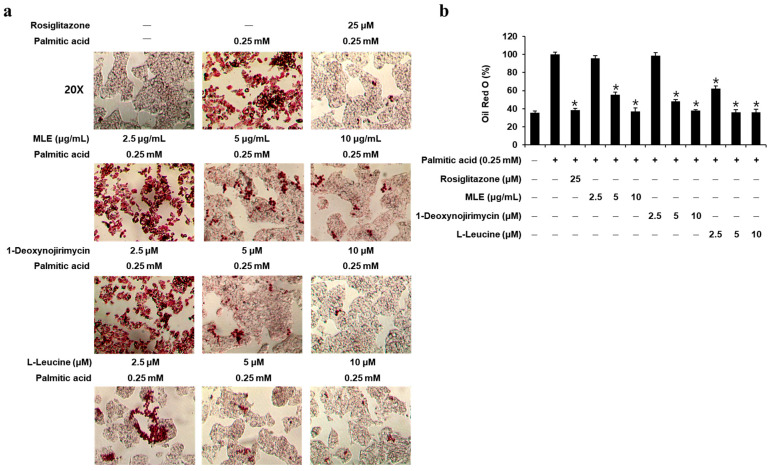
Inhibitory effects of mulberry leaf extract (MLE), 1-deoxynojirimycin (DNJ), L-leucine (LL), and rosiglitazone on lipid accumulation in HepG2 cells. (**a**) Images (×4 magnification) showing staining with ORO in HepG2 cells treated with PA (0.25 mM) for 24 h with or without pre-treatment with the indicated concentrations of MLE, DNJ, LL, and rosiglitazone. (**b**) Level of staining with ORO (*n* = 3 independent experiments, * *p* < 0.05, Kruskal–Wallis non-parametric test). Data are represented as the mean ± SEM.

To confirm the effect of MLE, DNJ, and LL on lipid accumulation at a non-toxic concentration, the cytotoxicities of MLE, DNJ, LL, and rosiglitazone (positive control) were evaluated in the HepG2 cells. The experiment for cell viability showed that the viability of HepG2 cells was unaffected by treatment with MLE, up to 10 μg/mL, DNJ, LL, and rosiglitazone, up to 10 µM (Figure S1). Thus, MLE of 2.5, 5, and 10 μg/mL, DNJ, LL, and rosiglitazone of 2.5, 5, and 10 μM were chosen for subsequent experiments in this study. In HepG2 cells, MLE (5, 10 μg/mL), DNJ (5, 10 μM), LL (2.5, 5, 10 μM), and rosiglitazone (25 μM) inhibited PA-induced lipid accumulation, as determined by staining with ORO (Figure 1). These results suggest that MLE, DNJ, and LL decrease PA-induced lipid accumulation in insulin-resistant HepG2 cells.

### 3.2. Inhibitory Effects of MLE, DNJ, and LL on the Amounts of IL-6 and MCP-1 in HepG2 Cells

**Figure 2:**
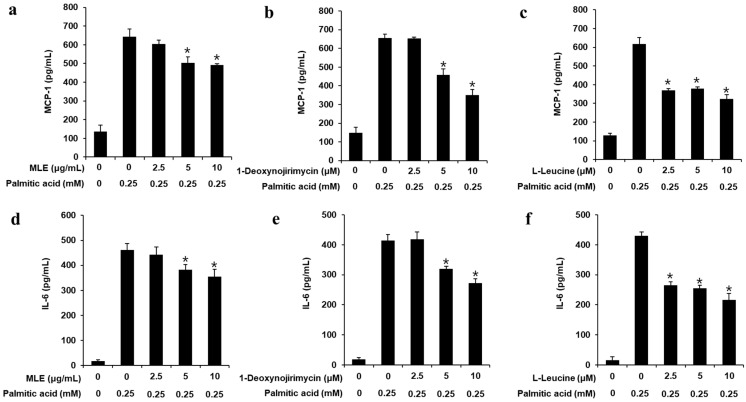
Inhibitory effects of mulberry leaf extract (MLE), 1-deoxynojirimycin (DNJ), and L-leucine (LL) on the amounts of interleukin (IL)-6 and monocyte chemoattractant protein (MCP)-1 in HepG2 cells. Pro-inflammatory cytokine amounts of MCP-1 in HepG2 cells treated with PA (0.25 mM) for 24 h either with or without the indicated concentrations of pre-treatment with (**a**) MLE, (**b**) DNJ, or (**c**) LL. Pro-inflammatory cytokine amounts of IL-6 in HepG2 cells treated with PA (0.25 mM) for 24 h either with or without the indicated concentrations of pre-treatment with (**d**) MLE, (**e**) DNJ, or (**f**) LL (*n* = 3 independent experiments, * *p* < 0.05, Kruskal–Wallis non-parametric test). Data are represented as the mean ± SEM.

Using ELISA kits, the amounts of MCP-1 and IL-6 in the cell culture supernatant were ascertained. MLE, DNJ, and LL prevented the synthesis of IL-6 and MCP-1 brought on by PA (Figure 2). These results suggest that MLE (5 and 10 μg/mL), DNJ (5 and 10 μM), and LL (5 and 10 μM) inhibit pro-inflammatory cytokine production in HepG2 cells treated with PA (0.25 mM).

### 3.3. Inhibitory Effects of MLE, 1-Deoxynojirimycin, and L-Leucine on the Protein Expression Levels of FAS and PPAR-γ in HepG2 Cells

**Figure 3:**
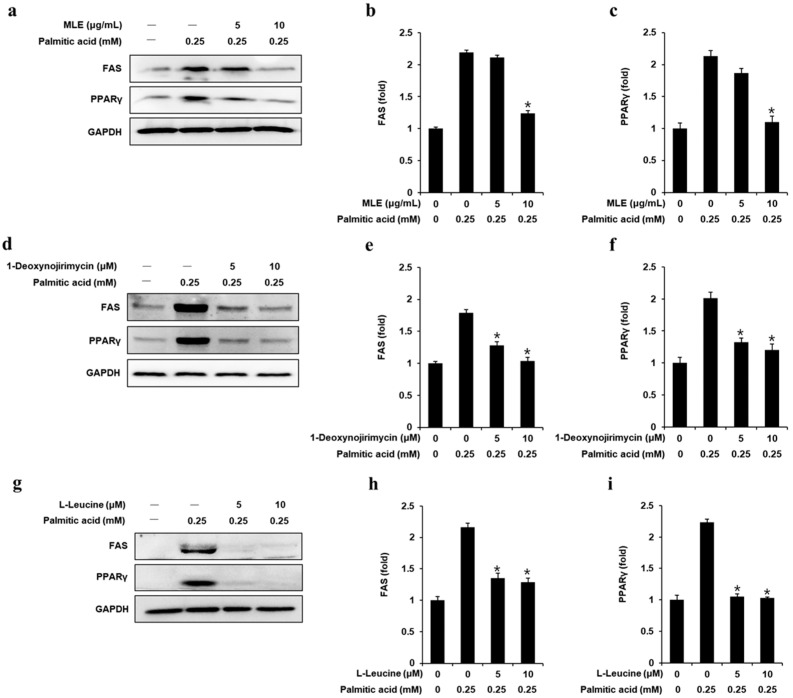
Inhibitory effects of mulberry leaf extract (MLE), 1-deoxynojirimycin (DNJ), and L-leucine (LL) on the expression levels of FAS and PPAR-γ in HepG2 cells. Ratios of FAS and PPAR-γ band intensities and protein expression levels in HepG2 cells treated with PA (0.25 mM) for 24 h, either with or without pretreatment with the specified doses of (**a**–**c**) MLE, (**d**–**f**) DNJ, and (**g**–**i**) LL (*n* = 3 independent experiments, * *p* < 0.05, Kruskal–Wallis non-parametric test). Data are represented as the mean ± SEM.

Using Western blotting, the protein expression levels of PPAR-γ and FAS were ascertained. According to Figure 3, 0.25 mM PA significantly increased the protein expression levels of FAS and PPAR-γ compared to those in untreated cells. This increase in expression was then inhibited following the addition of MLE (10 μg/mL), DNJ (5 and 10 μM), and LL (5 and 10 μM).

## 4. Discussion

**Figure 4:**
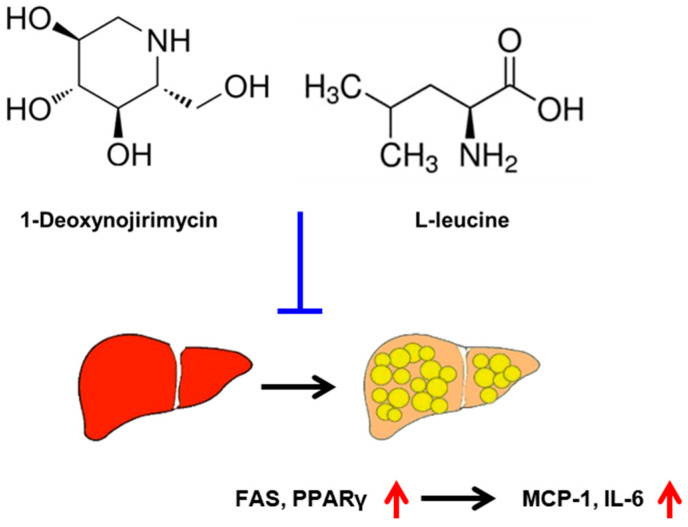
Schematic description on the therapeutic targets of 1-deoxynojirimycin (DNJ) and L-leucine (LL) in hepatic steatosis. The red arrow denotes upregulated gene expression.

A straight-chain saturated fatty acid called PA was chosen for this investigation in order to cause lipid accumulation in HepG2 cells. Exposure of HepG2 cells to PA led to an increase in lipid accumulation, which MLE, DNJ, LL, and rosiglitazone suppressed with similar effects. A commercially available water-based extract of the leaves of *M. alba* is MLE standardized to contain DNJ and LL. This investigation revealed that MLE, DNJ, and LL could inhibit FAS and PPAR-γ, suppressing lipid accumulation and pro-inflammatory cytokine levels in HepG2 cells. However, their precise mechanism of action in vitro remains to be elucidated. To the best of the author’s knowledge, the inhibitory effects of these compounds on hyperlipidemia in animal models and humans have been reported [6,7,8,9,11]. Rosiglitazone is an oral antidiabetic drug that exerts an inhibitory effect against PA-induced dyslipidemia in HepG2 cells [20] and amount of fat in the liver of people with type 2 diabetes [21]. Rosiglitazone was included as a positive control to provide a benchmark for the magnitude of lipid-lowering effects. Although rosiglitazone primarily functions as a PPARγ agonist, whereas MLE, DNJ, and LL appear to suppress PPARγ and FAS expression, its inclusion enables a meaningful comparative evaluation of anti-lipogenic efficacy under the same experimental conditions. Excessive triglyceride production in the liver stimulates macrophages, resulting in an inflammatory response [22]. Stimulated macrophages reportedly release IL-6 and MCP-1, which induce inflammation and interfere with carbohydrate and lipid metabolism, leading to hepatocyte hyperlipidemia [23,24]. In this study, MLE, DNJ, and LL reduced the PA-induced amounts of IL-6 and MCP-1, indicating their role in alleviating PA-induced inflammatory responses. Excessive dietary intake of free fatty acids activates enzymes associated with lipogenesis in hepatocytes, leading to excessive energy accumulation and triglyceride synthesis [25]. The expression of enzymes involved in lipid synthesis, including lipoprotein lipase (LPL) and FAS depends on binding a lipid transcription factor with the promoter region of lipid synthesis genes [26]. PPARγ is the main transcription factor for lipid synthesis. PA-induced activation of PPARγ is related to lipid accumulation in the liver of NAFLD mice [27], whereas that of FAS is related to lipid accumulation in the human normal hepatocytes [28]. Thus, PPARγ and FAS may be potential therapeutic drug targets to alleviate hepatic lipid accumulation (Figure 4). Based on our results showing the downregulation of PPARγ and FAS expression, it is plausible that MLE, DNJ, and LL may act as PPARγ antagonists or negative regulators. This potential mechanism could underlie their ability to reduce lipid accumulation and modulate lipogenic pathways in HepG2 cells. Further studies are warranted to confirm any direct interaction with PPARγ and to elucidate the precise molecular pathways involved.

In this study, we demonstrated that MLE, containing DNJ and LL, significantly attenuated PA-induced lipid accumulation and inflammatory responses in HepG2 cells. The choice of MLE was based on both historical and scientific evidence: mulberry leaves have been traditionally used in Asian medicine for metabolic disorders, and recent studies have confirmed their efficacy in improving hyperlipidemia, hepatic steatosis, and NASH in both animal models and human trials. Additionally, MLE offers a natural, safe source of potent α-glucosidase inhibitors and other bioactive compounds, which makes them a rational choice for investigating anti-lipogenic and anti-inflammatory effects in hepatocytes [6,7,8,9,10,11]. From a structure-activity perspective, DNJ is a natural amino sugar that mimics glucose and inhibits α-glucosidase, thereby reducing carbohydrate hydrolysis and postprandial glucose spikes. This glucose-lowering effect indirectly reduces lipogenesis by limiting substrate availability for de novo lipid synthesis [29]. LL, a branched-chain amino acid, activates the mTORC1 pathway, enhancing protein synthesis and improving insulin signaling, which can further regulate lipid metabolism in hepatocytes [30]. The combination of DNJ and LL may exert synergistic effects, with DNJ potentially reducing metabolic stress and LL possibly promoting insulin-mediated lipid utilization. Additionally, other polyphenols in MLE are likely to contribute antioxidant and anti-inflammatory activities, which could complement the effects of DNJ and LL. These proposed mechanisms are based on the known bioactivities of the individual components and serve as a hypothesis to explain the observed effects. Taken together, our findings suggest that MLE exerts its beneficial effects through multiple molecular mechanisms, including glucose-lowering, modulation of lipid metabolism, and anti-inflammatory actions, providing a mechanistic explanation for the improvements observed in both animal and human studies. Moreover, the PA-treated HepG2 cell model employed in this study effectively mimics key features of hepatic steatosis and inflammation, allowing for the evaluation of these molecular effects in a controlled in vitro environment. However, it is important to note that HepG2 cells are hepatoma-derived and may not fully replicate the lipid metabolism and inflammatory responses of normal hepatocytes. Therefore, while our findings provide valuable mechanistic insights, further validation in primary hepatocytes and in vivo models of NASH is necessary to confirm the translational relevance of MLE, DNJ, and LL.

## 5. Conclusions

As part of our ongoing investigation into bioactive constituents from diverse natural sources [31,32], we evaluated the effects of MLE and its active components, DNJ and LL, in PA-treated HepG2 cells and demonstrated that MLE, DNJ, and LL can reduce the expression of PPARγ and FAS. Therefore, our findings suggest that MLE, DNJ, and LL potentially reduce lipid accumulation by inhibiting PPARγ and FAS in HepG2 cells. These results indicate the potential of MLE, DNJ, and LL as candidates for preventing and treating hepatic steatosis.

## Data Availability

The original contributions presented in this study are included in the article/Supplementary Materials. Further inquiries can be directed to the corresponding authors.

## Supplementary Materials

The following are available online at https://www.mdpi.com/article/10.3390/biomedicines13122930/s1; Figure S1: Effects of mulberry leaf extract (MLE), 1-deoxynojirimycin (DNJ), and L-leucine (LL) on the HepG2 cell viability; Figure S2: LC–MS chromatographic analysis of mulberry leaf extract (MLE).
