# Supplementary material for: Effect of Mulberry Leaf and Its Active Component, 1-Deoxynojirimycin, on Palmitic Acid-Induced Lipid Accumulation in HepG2 Cells
Source: Biomedicines. 2025 Nov 28;13(12):2930. doi: 10.3390/biomedicines13122930 (PMC12730792; doi:10.3390/biomedicines13122930)
Supplement: Supplementary file 1 [file biomedicines-13-02930-s001.zip › biomedicines-3968868-supplementary.pdf]

Supplementary data

Effect of Mulberry Leaf and Its Active Component, 1-deoxynojirimycin on Palmitic Acid-Induced Lipid Accumulation in HepG2 Cells

Dahae Lee [1,†], Jiyeon Kim [1,†], Min Ji Han [2], Seon Hwa Kim [2], Tae Hoon Kim [2], Dae-Woon Eom [3], Inhyeok Song [4], Daesik Jeong [4,5], Noriko Yamabe [4,*] and Ki Hyun Kim [1,*]

[1]*School of Pharmacy, Sungkyunkwan University, Suwon 16419, Republic of Korea; pjsldh@gachon.ac.kr (D.L.); delucete@naver.com (J.K.)*

[2]*Vixxol Corporation, Gunpo 15807, Republic of Korea; minjhan@vixxol.com (M.J.H.); seonhkim@vixxol.com (S.H.K.); andykim@vixxol.com (T.H.K.)*

[3]*Department of Pathology, University of Ulsan College of Medicine, Gangneung Asan Hospital, Gangneung, 210-711, Republic of Korea; edwjyh@hanmail.net (D.-W.E.)*

[4]*Bio DX Group, InSiliCox, Seoul 03016, Korea; inhyeok@5works.co.kr (I.S.); jungsoft97@smu.ac.kr (D.J.)*

[5]*Faculty of SW Convergence, Sangmyung University, Seoul 03016, Republic of Korea; akinom949@gmail.com (M.R.)*

[†]These authors contributed equally to this study.

* Corresponding authors:

Ki Hyun Kim, Tel: +82-31-290-7700; Fax: +82-31-290-7730; E-mail: khkim83@skku.edu

Noriko Yamabe, E-mail: Nonyama77350@msn.com

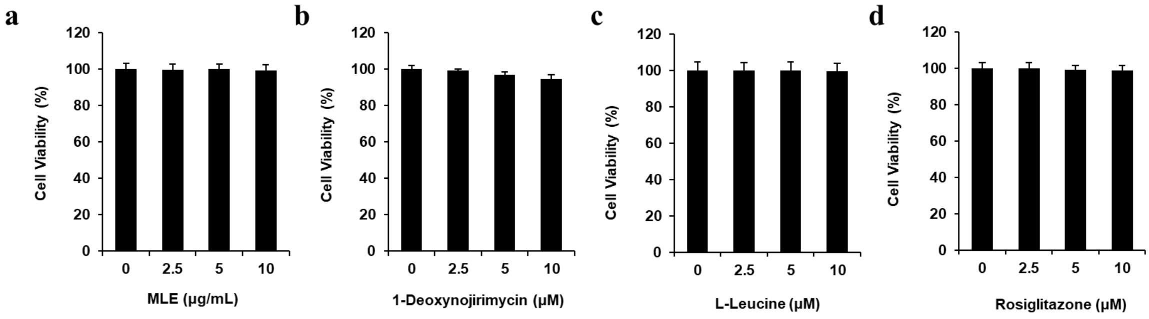

Figure S1. Effects of mulberry leaf extract (MLE), 1-deoxynojirimycin (DNJ), and L-leucine (LL) on the HepG2 cell viability. HepG2 cell viability after treatment with (**a**) MLE, (**b**) DNJ, (**c**) LL, and (**d**) rosiglitazone at 0, 2.5, 5, and 10 µM for 24 h, as determined by the Ez-Cytox cytotoxicity assay (n = 3 independent experiments). Data are represented as the mean ± standard error of the mean (SEM).

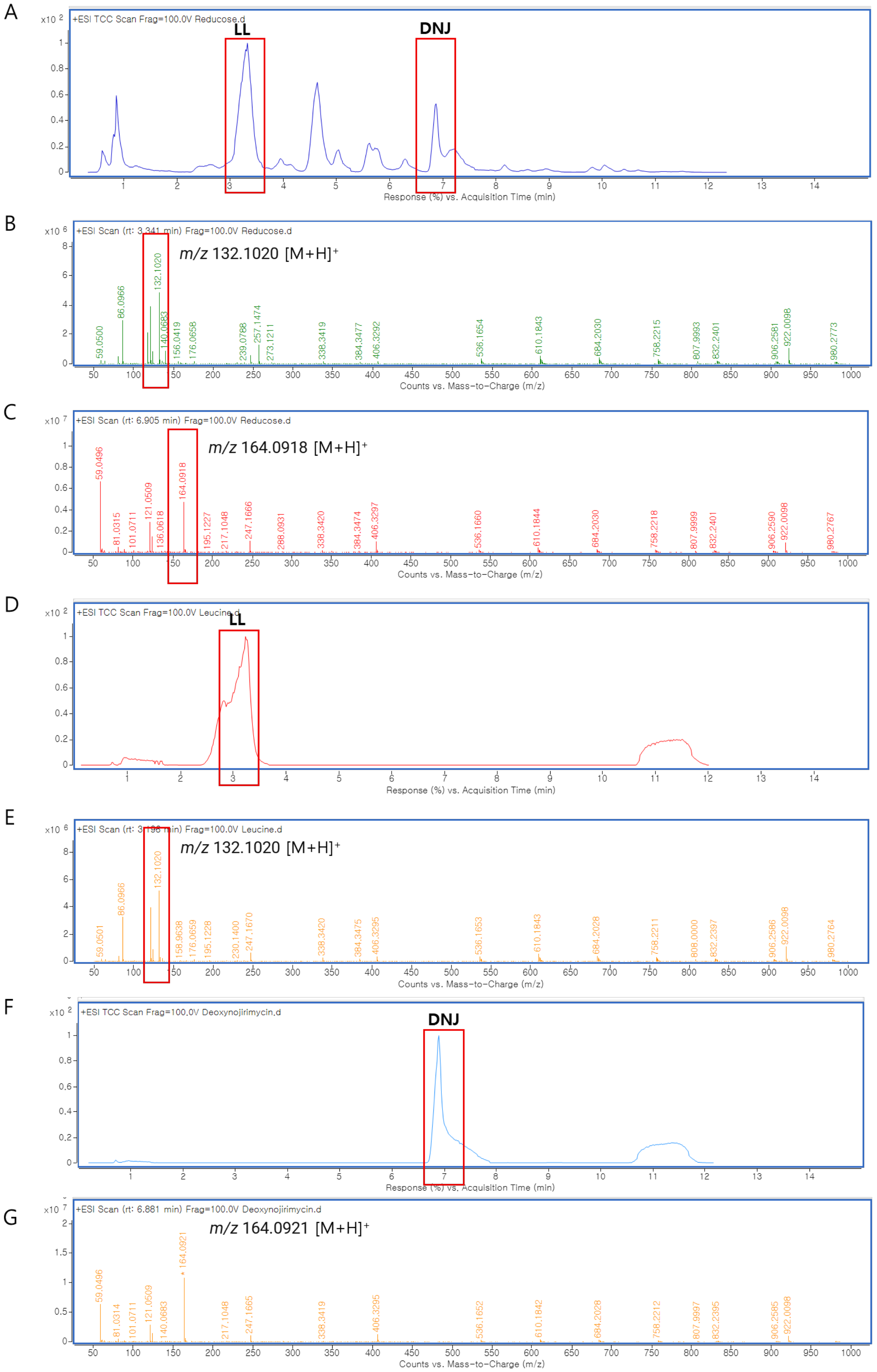
A
+ESI TCC Scan Frag=100.0V Reducose.d
LL
DNJ
Response (%) vs. Acquisition Time (min)
B
+ESI Scan (rt: 3.341 min) Frag=100.0V Reducose.d
m/z 132.1020 [M+H]+
Counts vs. Mass-to-Charge (m/z)
C
+ESI Scan (rt: 6.905 min) Frag=100.0V Reducose.d
m/z 164.0918 [M+H]+
Counts vs. Mass-to-Charge (m/z)
D
+ESI TCC Scan Frag=100.0V Leucine.d
LL
Response (%) vs. Acquisition Time (min)
E
+ESI Scan (rt: 3.196 min) Frag=100.0V Leucine.d
m/z 132.1020 [M+H]+
Counts vs. Mass-to-Charge (m/z)
F
+ESI TCC Scan Frag=100.0V Deoxynojirimycin.d
DNJ
Response (%) vs. Acquisition Time (min)
G
+ESI Scan (rt: 6.881 min) Frag=100.0V Deoxynojirimycin.d
m/z 164.0921 [M+H]+
Counts vs. Mass-to-Charge (m/z)

Figure S2. LC–MS chromatographic analysis of mulberry leaf extract (MLE). (A) Total compound chromatogram (TCC) of the mulberry leaf extract (MLE). (B) ESI–MS spectrum of the peak at 3.3 min corresponding to leucine (LL) in MLE. (C) ESI–MS spectrum of the peak at 6.9 min corresponding to 1-deoxynojirimycin (DNJ) in MLE. (D) Total compound chromatogram (TCC) of the LL standard. (E) ESI–MS spectrum of the 3.3-min peak from the LL standard. (F) Total compound chromatogram (TCC) of the DNJ standard. (G) ESI–MS spectrum of the 6.9-min peak from the DNJ standard.

LC–MS (Q-TOF) Analysis of Mulberry Leaf Extract

The chemical composition of the mulberry leaf extract (MLE) was analyzed using an Agilent 1290 Infinity II HPLC system coupled to an Agilent G6545B quadrupole time-of-flight (Q-TOF) mass spectrometer. Chromatographic separation was achieved on a Poroshell 120 HILIC-Z column (2.1 × 100 mm, 2.7 µm; Agilent Technologies, USA) using a mobile phase consisting of 0.1% formic acid in water (A) and 0.1% formic acid in acetonitrile (B) under a gradient elution program. A gradient elution was applied starting at 5% A and 95% B, maintained for 0.1 min to focus analytes at the column head. From 0.1 to 10 min, the proportion of solvent A was linearly increased from 5% to 25%, while solvent B decreased from 95% to 75% to facilitate the elution of highly polar compounds such as DNJ and LL. The gradient was then rapidly returned to initial conditions (5% A, 95% B) between 10.0 and 10.1 min and held until 15 min for proper column re-equilibration. The flow rate was maintained at 0.4 mL/min, the column temperature at 30 °C, and the injection volume at 2 µL. The mass spectrometer was operated with a drying gas flow of 8 L/min at 325 °C, a fragmentor voltage of 70 V, a capillary voltage of 3500 V, and a mass scan range of *m/z* 50–1000.

MLE was analyzed at 130 ppm, while authentic standards of DNJ (12 ppm) and leucine (10 ppm) were included for identification. LC–MS analysis confirmed the presence of DNJ and identified L-leucine (LL) in the extract based on retention time matching and accurate mass measurement. The corresponding chromatograms and spectra are provided in Figure S2.
